# Adult-Onset Linear Morphea (*en coupe de sabre*) of the Face Successfully Treated with Photoactivated Low-Temperature Platelet-Rich Plasma: A Valid Therapeutic Option

**DOI:** 10.3390/medicina59061114

**Published:** 2023-06-09

**Authors:** Santo Raffaele Mercuri, Matteo Riccardo Di Nicola, Vittoria Giulia Bianchi, Giovanni Paolino

**Affiliations:** 1 Unit of Dermatology and Cosmetology, I.R.C.C.S. San Raffaele Hospital, 20132 Milan, Italy; mercuri.santoraffaele@hsr.it (S.R.M.); vittoriaabianchi@gmail.com (V.G.B.); paolino.giovanni@hsr.it (G.P.); 2 Medicine and Surgery Faculty, San Raffaele Vita-Salute University, 20132 Milan, Italy

**Keywords:** morphea, linear, *en coupe de sabre*, MCT Plasma, scleroderma, platelet rich plasma, PRP, therapy, treatment

## Abstract

Localized scleroderma (also known as morphea) is a chronic autoimmune disorder characterized by depressed, fibrotic, and dyschromic cutaneous lesions. It has a significant impact on the patient’s daily life due to the unaesthetic evolution of the cutaneous lesions. Morphea is clinically divided into linear, circumscribed (plaque), generalized, pansclerotic, and mixed forms. Linear morphea *en coupe de sabre* (LM) usually arises in childhood. However, in about 32% of cases, it may arise in adulthood, showing a more aggressive course with also an increased risk of systemic involvement. Methotrexate is the first-line treatment for LM, although systemic steroids, topical agents (corticosteroids and calcineurin inhibitors), hyaluronic acid injections, and hydroxychloroquine or mycophenolate mofetil are valid therapeutic options. In any case, these treatments are not always effective and sometimes can be associated with important side effects and/or not tolerated by the patients. In this spectrum, platelet-rich plasma (PRP) injection can be considered a valid and safe alternative since PRP injections in the skin induce the release of anti-inflammatory cytokines and growth factors, thus reducing inflammation and increasing collagen remodeling. Herein, we describe a successful treatment of an adult-onset LM *en coupe de sabre* with photoactivated low-temperature PRP (Meta Cell Technology Plasma) sessions, showing an important local improvement of the lesion and patient satisfaction.

## 1. Introduction

Localized scleroderma (also known as morphea) is a cutaneous chronic and autoimmune disorder characterized by fibrosis of the skin with a higher incidence in female patients, causing cutaneous morpho-structural alterations with considerable aesthetic impact in the daily life of patients. Morphea can show different clinical presentations. Indeed, it is usually divided into linear, circumscribed (plaque), generalized, pansclerotic, and mixed forms [1,2,3]. Linear morphea (LM) mainly involves the head/neck region and usually arises during childhood; however, in some cases, LM may appear for the first time in adulthood, showing a more aggressive topical and systemic involvement and therefore requiring prompt treatment. It is known that treatment of morphea is often a challenge for clinicians, given the poor therapeutic response of the lesions. Systemic and topical steroids, methotrexate, topical calcineurin inhibitors (pimecrolimus and tacrolimus), ultraviolet-A1, and (although rarely) topical hyaluronic acid injections, hydroxychloroquine, or mycophenolate mofetil are usually prescribed as main therapeutic options, albeit with different and limited results [1,2,3]. When these therapeutic options are not effective, not tolerated, and/or not accepted by the patients, alternative therapies can be proposed.

Although underreported in the literature, platelet-rich plasma (PRP) treatment can be a valid therapy in some patients. PRP is mainly characterized by a plasma fraction taken from peripheral blood that, once injected into cutaneous lesions, can release several anti-inflammatory cytokines and growth factors, which perform the remodeling of the collagen bundles, also via mitogen activity [4,5]. Herein, we describe a successful treatment of a linear morphea *en coupe de sabre* with Meta Cell Technology (MCT) plasma treatment, a new photothermal-activated PRP.

## 2. Case Report

**Figure 1:**
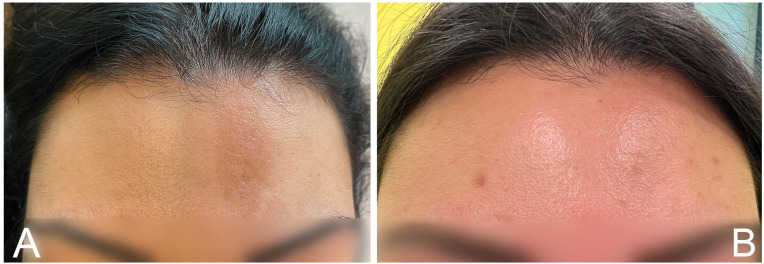
Linear morphea in frontal area of the 25-year-old female patient before (**A**) and after (**B**) 10 injections of MCT plasma treatment.

A 25-year-old female patient presented at our hospital with an 18-month-old history of linear morphea (*en coupe de sabre*) in the frontal area (Figure 1A). Specifically, she had a band of non-tender, non-erythematous sclerotic skin over the frontal area, with a brownish appearance. Her personal medical record included insulin resistance and polycystic ovary, and she was under metformin treatment. Standard blood parameters were found in the normal range, including antinuclear, anti-double-stranded DNA, anticentromere, anti-RNP, and anti-Scl-70 antibodies. For the cutaneous lesions, the patient received topical steroid treatments (2 months), topical pimecrolimus (10 months), and systemic methotrexate (12 months) without any clinical improvement. Therefore, after written and informed consent, we proposed and started MCT plasma sessions.

After local disinfection, 11 milliliters of the patient’s blood were collected from the peripheral cubital forearm vein using a standard 21 G butterfly needle and transported in 3.8% Na Citrate vacutainers, which separate red blood cells from the plasma containing the buffy coat. Then, vacutainers were centrifuged at room temperature for 10 min at 1500 rpm (Tropocells^®^ PRP kit (Estar Medical, Holon, Israel)). We obtained 4 mL of PRP, resulting in a three-fold platelet count.

Before injecting PRP into the patient, we took an additional step using a new technology called MCT^®^ Plasma (Meta Cell Technology, Sant Cugat del Valles, Barcelona, Spain). Specifically, the collected PRP was transferred to a special 10 mL medical container (MCT Kit^®^, Barcelona, Spain) and then inserted into a photothermal stimulator machine (MCT Unit^®^, Barcelona, Spain) that provides controlled 4 ^°^C and a 623.5 nm red light source. Light absorption in the electron transport chain of mitochondrial platelets leads to the acceleration of electron transfer reactions and ATP production. The process increases intracellular calcium concentration (Ca^2+^) and intercellular calcium mobilization with a final increased release of growth factors. At the same time, it primes platelets for activation, with a further increased release of VEGF, EGF, and b-FGF in a flash but also in a sustained way. This process of photothermal activation takes 15 min. Finally, PRP was placed in 1 mL Luer-Lock syringes with a 27 g needle for subcutaneous injection.

The patient performed five sessions (in which PRP was injected directly over the LM), one every 2 months. No adverse reactions have been observed except mild pain during the injection. After the first month of treatment, a slight improvement was observed; an improvement close to 100% of the whole lesion was evident after five injections, with high patient satisfaction and a positive impact on her daily life (Figure 1B—photographic assessment done independently by two dermatologists collaborating in our unit: A.F. and F.V.). After a follow-up of 6 months, the cutaneous improvement is still present, without any sign of local and/or systemic recurrence. She continues to perform one session of MCT plasma every 6 months and to perform periodic clinical and instrumental investigations. The patient is very satisfied with the response to the treatment.

## 3. Discussion

Compared to child-onset, adult-onset LM is poorly studied, although it occurs in 32.6% of patients with LM [2]. Adult-onset LM may present an aggressive course also involving extra-cutaneous sites with systemic symptoms and a more aggressive local and systemic course. Therefore, in this type of patient, each therapeutic option must be carefully evaluated and carried out. In this regard, systemic therapy is usually the therapeutic gold standard for the treatment of LM; specifically, methotrexate (MTX) is considered the first-line therapy in adult- and child-onset LM, resulting in resolution, reduction in progression, and reactivation of the lesions. Other treatments include systemic steroids and topical treatments (such as steroids and calcineurin inhibitors); however, these therapies often show long-term side effects, such as atrophy with topical steroids and increased risk of secondary infections and/or infective reactivation with calcineurin inhibitors.

Autologous PRP is characterized by a high platelet and growth factor concentration. Specifically, PRP promotes the release of Platelet-Derived Growth Factor (PDGF), Vascular Endothelial Growth Factor (VEGF), Transforming Growth Factor (TGF) type α and β, Connective Tissue Growth Factor (CTGF), and anti-inflammatory cytokines, such as Interleukin (IL)-1ra (IL-1 antagonist receptor), soluble tumor necrosis factor receptor (s-TNFr) and IL-10 [4]. All these growth factors and anti-inflammatory cytokines may stimulate new collagen production and tighten/rearrange existing collagen fibers, allowing its remodeling [1]. PRP also contains some glycoprotein of the extracellular matrix, such as fibronectin, vitronectin, fibrinogen, osteocalcin, and osteonectin, that may activate the extracellular matrix constitution [1]. Besides, PRP may stimulate collagen and, by reducing the COX2 and CXCR4 gene expression, it plays a big role in reducing chronic inflammation, thus justifying its use in scars [1,5]. After the injection of PRP, platelets continue to synthesize and secrete proteins until their life span (about 10 days).

Regarding the existing types of PRP, different authors have tried to categorize them, but there is still no uniquely recognized classification that allows us to accurately compare the effectiveness of different devices and different studies [6,7,8]. An often-used classification is the one of Dohan Ehrenfest et al. [6,7,8], which proposes a division into the following four categories based on the presence/absence of cell content and the fibrin architecture:

(a) Pure platelet-rich plasma: a PRP poor in leukocytes, with a low-density fibrin network after activation;
(b) Leukocyte-rich platelet-rich plasma: a PRP rich in leukocytes with a low-density fibrin network after activation;
(c) Pure platelet-rich fibrin: a PRP poor in leukocytes, with a high-density fibrin network (unlike the previous ones, this cannot be injected and exists in an activated gel form);
(d) Leukocyte- and platelet-rich fibrin: a PRP rich in leukocytes with a high-density fibrin network.

To these, a fifth type can be added: the leukocyte-rich platelet-rich plasma with a relatively high concentration of monocytes (monocyte-rich PRP; see [6] for further details).

The use of the MCT method, transforming PRP to MCT plasma with light and temperature, allowed us to achieve better results, allegedly due to a further increase in the release of growth factors.

Photo-modulation action is a very well-documented process that plays a major role in increasing cell reactivity and efficiency. In its interaction with mitochondrial photo-acceptors, light impacts ATP synthesis, growth factor release, kinase activity, intracellular cation concentration, and retrograde signaling.

Indeed, with photoactivation there is a light absorption of cytochrome C oxidase (COX), leading to the acceleration of electron transfer reactions and ATP production and relative increase intracellular calcium concentration (Ca^2+^); consequently, the increase in intracellular Ca^2+^ in platelets induces integrin alphaIIß3 activation, granule release (VEGF, EGF, PDGF, bFGF, and TGF-ß) aggregation, and thrombus formation [9,10,11].

Currently, cases of LM (*en coupe de sabre*) treated with PRP are underreported; to date, Belgaumkar et al. [1] reported a case of a 24-year-old female patient with an en coup de sabre LM successfully treated with conventional PRP, while more recently, Wang et al. [12] described the case of a 31-year-old woman with an LM of the face, successfully treated with an autologous growth factor concentration. In both cases, the patients showed a clear clinical improvement of the skin lesions without the onset of specific adverse reactions. Virzì et al. [13] proposed a therapy consisting of a combined PRP and lipofilling treatment in a sample of six patients with scleroderma, showing an improvement in the clinical signs. However, in their sample, no patients had LM on their faces. At the same time, Pirrello et al. [14] used hyaluronic acid and PRP for the treatment of microstomia in scleroderma patients with good results and stability up to 2 years of follow-up. Finally, Ibrahim et al. [15] conducted (on five morphea patients) weekly sessions of PRP for a total of 12 sessions, with a general improvement and only mild discomfort during the procedure. However, among all these studies listed in the literature, only one case of LM (*en coupe de sabre*) of the face treated with PRP was reported [1], underlining how this therapeutic option is still little applied in daily clinical practice for this condition.

## 4. Conclusions

LM is an autoimmune disease with a predominantly cutaneous localization and possible systemic involvement that is often associated with cutaneous disfigurations and a significant impact on the daily life of patients, being a social stigma. The treatment is mainly characterized by topical creams and/or systemic therapy. However, these treatments are not always effective, tolerated, and/or accepted by patients. For this reason, it is important to be able to offer valid therapeutic alternatives. In this spectrum, MCT Plasma is a promising, effective, and well-tolerated treatment for patients with LM *en coupe de sabre*, with a low risk of side effects and easy to perform. Ultimately, LM *en coupe de sabre* is not a frequent condition, and treatment with PRP and MCT plasma is still little used. Further prospective studies are needed to better investigate the role and efficacy of PRP and MCT plasma in LM to also have more cases.

## Data Availability

No new data were created or analyzed in this study. Data sharing is not applicable to this article.
